# Assessment of Allelopathic Potential of Cotton Chromosome Substitution Lines

**DOI:** 10.3390/plants13081102

**Published:** 2024-04-15

**Authors:** Worlanyo Segbefia, Varsha Singh, Mary Gracen Fuller, Ziming Yue, Fernanda Reolon de Souza, Te Ming Tseng

**Affiliations:** Department of Plant and Soil Sciences, Mississippi State University, 32 Creelman St., Starkville, MS 39762, USA; ws788@msstate.edu (W.S.); vv219@msstate.edu (V.S.); maf572@msstate.edu (M.G.F.); zy101@msstate.edu (Z.Y.); fr278@msstate.edu (F.R.d.S.)

**Keywords:** chromosome substitution, lines of cotton, allelopathy potential, weed control

## Abstract

Weed interference consistently poses a significant agronomic challenge in cotton production, leading to unfavorable direct and indirect consequences. Consequently, the predominant strategy employed to manage weeds is the application of synthetic herbicides. However, this extensive reliance has resulted in the development of herbicide-resistant weed populations due to the prolonged use of a single herbicide and the lack of rotation. This project focused on identifying weed-suppressive cotton chromosome substitution (CS) lines. These CS lines closely resemble the parent TM-1, an upland cotton derivative (*Gossypium hirsutum*). Each CS line carries a single chromosome or chromosome arm exchanged from *G. barbadense*, *G. tomentosum*, or *G. mustelinum* within the TM-1 background. In a greenhouse experiment utilizing a stepwise approach, five CS lines, along with two conventional varieties (Enlist and UA48) and the parent line (TM1), were assessed to determine their potential for suppressing Palmer amaranth growth. The plant height was measured 7, 14, and 21 days after establishment, and the chlorophyll content was measured 21 days after establishment. The results revealed varying levels of chlorophyll reduction in Palmer amaranth, with the Enlist variety displaying the lowest reduction (32%) and TM-1 exhibiting the highest (78%). Within 14 days of establishment, the CS lines T26lo, BNTN 1-15, and T11sh demonstrated substantial suppression of Palmer amaranth height, with reductions of 79, 70, and 71%, respectively. Conversely, Enlist displayed the least effective performance among the CS lines. Moreover, CS22, CS49, CS50, CS34, UA48, and CS23 displayed a decreasing trend in reducing Palmer amaranth height from 14 to 21 days after establishment. This research demonstrates the inherent herbicidal attributes within cotton CS lines against Palmer amaranth. In light of the versatile applications of cotton fibers and the unique characteristics of the *G. hirsutum* genome, this study investigates the potential of specific cotton lines in enhancing weed management practices. By elucidating the implications of our findings, we aim to contribute to promoting sustainability and developing alternatives to synthetic herbicides in agricultural systems.

## 1. Introduction

Cotton (*Gossypium* L.) is a vital summer annual crop in warm temperate regions, is grown in more than 70 countries around the globe, and has a share of around 31% in the world fiber market [1]. Providing approximately 35 percent of global cotton exports in recent years, the United States is the world’s leading cotton exporter. Among the U.S. States, Texas is the largest producer, accounting for approximately 40 percent of U.S. cotton production, with other top producers being Georgia, Mississippi, and Arkansas [2]. The plant is also a known source of valuable chemical compounds, including fatty acids, lipids, carbohydrates, and phenolics [2]. Cotton fibers produce various consumer goods, including oils, paper products, animal feed, and textiles [3]. The genome of *Gossypium hirsutum* is homogeneous and big (2n = 52) [4]. Minimal genetic variation exists in the genome due to *G. hirsutum* cultivars’ domestication and constant inbreeding. Herbicide-resistant weed populations are one of the agricultural issues that farmers, geneticists, and weed scientists have limited material to work with due to genetic variation. Saha et al. created chromosomal substitution (CS) cotton lines in 2006 using interspecific introgression. The genome of *G. hirsutum* contains chromosomes from three other tetraploid cotton species: *G. tomentosum*, *G. barbadense*, and *G. mustellinum* [4].

Weeds are frequently found in crop plant fields, and reduce crop yields, raise production costs, and reduce the cost-effectiveness of crop production [3]. Ref. [5] claim that weeds in agricultural areas reduce the quantity and quality of agricultural goods, causing farmers to suffer significant financial losses. The losses from weeds exceed those brought on by any type of agronomic pest, including rodents, nematodes, insects, diseases, and nematodes [6]. Weeds have been shown to reduce agricultural productivity by 34% on average [7]. According to [7], the following commercial crops exhibit harvest decreases as a result of weeds: wheat (23%), potatoes (30%), cotton (36%), rice (37%), soybeans (37%), and maize (40%).

Palmer amaranth (PA) and other weeds in cotton fields have historically been controlled by suitable cultivation, pre-planting, and timed herbicide applications (pre-emergence and postemergence herbicide application) [2,8]. Herbicides, often known as weedkillers, are pesticides used to manage undesirable plants in fields such as agriculture, forestry, gardening, and landscaping [9,10,11]. According to [12], the World Health Organization categorized glyphosate in 2015 as probably carcinogenic to humans. Palmer amaranth was identified by the Weed Science Society of America (WSSA) as the most troublesome plant in cotton fields nationwide in 2017. Controlling and managing weed invasion in the western United States rangelands of the U.S. Department of Agriculture cost USD 2.5 billion per year during the 1960s [13] and USD 340 million per year in 1989 in seventeen western United States. In addition, approximately USD 4 billion is spent annually on weed control through weedicides/pesticides [14]. In their report of 2021, ref. [15] reported that, as an essential aspect of weed management, it was better to prevent weeds from spreading than wait for their maturity and consequent damage before controlling them.

Allelopathy is an ecological phenomenon whereby certain organisms have a positive or negative impact on the functioning of other organisms nearby [16]. Allelochemicals is a term used to describe the substances created during allelopathy. These secondary metabolites, which include alkaloids, terpenoids, flavonoids, jasmonates, glucosinolates, amino acids, phenolics, momilactone, carbohydrates, salicylates, and hydroxamic acid [17], are released by plants through volatile exudation, leaf leachate, or root secretion. Plant-based herbicides, as opposed to synthetic ones, have recently received more attention [18]. Extensive research has been conducted on crop varieties such as rice (*Oryza sativa*), wheat (*Triticum aestivum*), sunflower (*Helianthus annuus*), sweet potato (*Ipomoea batatas* (L.) Lam.), and canola (*Brassica napus*), revealing their potential to exhibit weed-suppressive traits. However, the allelopathic effects of cotton have remained relatively unexplored. In light of this, the current study seeks to investigate the weed-suppressive capabilities of cotton chromosome substitution lines (CS lines) through a greenhouse experiment involving a prevalent and troublesome cotton weed, *Palmer amaranth*.

## 2. Materials and Methods

**Figure 1:**
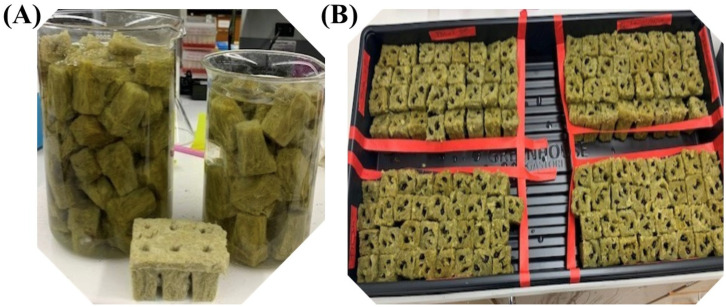
Preparation of rockwool for seed germination and growth; (**A**) rockwool soaked in pH-adjusted distilled water and 5% acetic acid for approximately 30 min to enhance absorption; (**B**) seedlings planted in treated rockwool and maintained in a growth chamber at a humidity level of 53%, day/night temperature, and cycle of 16/8 h and 28/24 °C, respectively.

**Figure 2:**
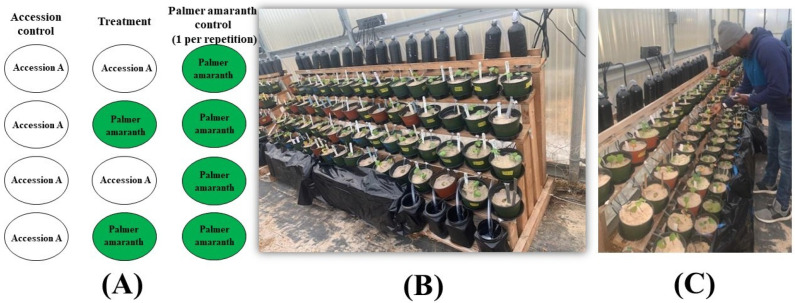
Illustration of the experimental setup within the stairstep structure: (**A**) The arrangement of experimental units featuring columns designated for treatments, accession controls, and Palmer amaranth controls (green circles). Each cotton line under examination has two distinct columns: the treatment and the control column. The control column comprises four consecutive pots of the same cotton line. On the other hand, the treatment column consisted of four pots where two pots containing the same cotton line alternated with two pots containing PA. This design was replicated for each cotton line tested, and for each repetition, there was a single column of four pots containing PA that served as a weed control column; (**B**) visualization of the stairstep layout with the organized distribution of experimental units. Each collection tank is equipped with a pump for water distribution through tubing; (**C**) the data collection process, encompassing evaluations of plant height on days 7, 14, and 21 after establishment (DAE) and chlorophyll concentration at 21 DAE.

This greenhouse experiment was conducted at the R.R. Foil Plant Science Research Center, Mississippi State University, from November 2020 to March 2021. Eight cotton lines that included five chromosome substitution (CS) lines, two conventional varieties, i.e., Enlist and UA48, and the parent (TM-1) were utilized for this project. The five CS lines comprised CS23, CS22, CS49, CS50, and CS34. These lines were formed by replacing corresponding pairs of chromosomes of *Gossypium hirsutum* (TM-1) with those of *Gossypium barbadense* (CS-B), *Gossypium tomentosum* (CS-T), and *Gossypium mustellinum* (CS-M). The weed suppressiveness of the cotton lines (donor species) was screened against a recipient plant, Palmer amaranth (*Amaranthus palmeri*). While the cotton seeds were hand-harvested from the field, Palmer amaranth seeds were bought from Azlin Seed Services (Leland, MS 38756). The cotton seeds with fiber were acid-washed in sulphuric acid, baking soda, and water. They were sun-dried in the greenhouse for a week. Rockwool was soaked in distilled water and 5% acetic acid, with a pH of 5.0–6.0, for 30 min (Figure 1). The cotton seeds and PA seeds were germinated in rockwool and kept in a growth chamber for 1–2 weeks, and a greenhouse top was used to cover the tray to maintain the humidity. The growth chamber was configured with a humidity level of 53%, day/night temperature, and cycle of 16/8 h and 28/24 °C, respectively. When the seedlings had fully established (attained two leaves), two cotton seedlings and three Palmer amaranth seedlings each were transplanted into pots of Quickrete Play Sand (silicon dioxide). These pots were kept in the greenhouse, maintaining the same temperature, humidity, and day/night cycle conditions as those in the growth chamber, for two weeks, for the seedlings to establish. Each of the pots represented an experimental unit. After establishment, these pots were placed onto a stairstep structure following the guidelines provided by [19]. The experiment was carried out with a Completely Randomized Design, with three replications in the greenhouse, and was repeated three times (Figure 2).

Six rows in each column made up the stairstep structure of the experimental setup. The bottom step held a collecting tank with a pump, while the top held a bottle. Pots of size 15 × 10 cm (diameter × depth) containing experimental units were placed on the middle four steps. A control column and a treatment column for each cotton line were included in the arrangement. Four rows in the control column contained one pot with two plants of the same cotton line. Two pots of the same cotton line and two pots of Palmer amaranth were arranged in alternate rows in the treatment column. One column of pots per repetition containing only Palmer amaranth seedlings was used as a weed control column. Plastic tubing attached to the pumps in the collecting tanks ran from the bottles on the top step to the appropriate collecting tank of each column.

The pumps in each collecting tank were set on a timer, which turned them on every six hours, and water from the collection tank was pumped to the bottles on the top step, where it trickled down to the pots and finally made its way back to the tank. Since we were using distilled water throughout the experiment, Hoagland’s No. 2 basal salts were added to the collecting tank every two weeks after establishment to avoid nutrient deficiencies. Each column had its bottle, pump, collecting tank, and tubing, making it a closed-loop system. At 7, 14, and 21 days after establishment (DAE), the height of all the plants was recorded using a meter rule. The chlorophyll content of both cotton and weed seedlings was also recorded using a hand-held CCM300 chlorophyll meter (Opti-Science, Hudson, NH, USA).

Palmer amaranth height and chlorophyll reduction were calculated using the following formulae:

$$Height\;reduction(\%)=\left[\frac{height\;of\;control\;PA\left(cm\right)-height\;of\;treatment\;PA(cm)}{height\;of\;control\;PA(cm)}\right]\times 100$$

$$Chlorophyll\;concentration\left[cci\right]reduction\left(\%\right)=\left[\frac{cc\;of\;control\;PA\left(cci\right)-cc\;of\;treatment\;PA(cci)}{cc\;of\;control\;PA(cci)}\right]\times 100$$

Data were analyzed using R software (version R—4.3.3) at *p* ≤ 0.05. Next, hierarchal clustering was applied to visualize the correlation among and between variables and components in R software. This technique clustered the cotton lines into associations based on Palmer amaranth inhibition (height and chlorophyll concentration). Data for height reduction values at 14 and 21 DAE, chlorophyll reduction at 21 DAE, and mean susceptibility were analyzed separately. Mean values were separated using Fisher’s Protected LSD at a probability level of 0.05 in JMP 14 (JMP^®^, Version 13, SAS Institute Inc., Cary, NC, USA, 1989–2007).

## 3. Results

The suppression of the PA by the cotton lines under study was calculated based on the impact of these lines on the height and chlorophyll reduction and mean susceptibility of PA (Table 1). The cotton lines were plotted against the height and chlorophyll reduction (%) of Palmer amaranth. At 7 DAE, there was no significance in the PA mean height reductions. The PA’s mean height reduction (%) at 21 DAE was the least for Enlist and the highest for TM1. Meanwhile, the mean chlorophyll content (cci) reduction for PA was the lowest for Enlist and the highest for CS34. At the end of the 21 days, the worst-performing variety was Enlist, and the best was CS line CS34, in relation to PA’s mean chlorophyll and PA mean height reduction (%) at 21 DAE.

At 14 DAE, the commercial cultivar UA48 demonstrated a 67% reduction in Palmer amaranth (PA) height, compared to a 55% reduction at 21 DAE. UA48 outperformed (66%) CS23 (62%) at 14 DAE, while at 21 DAE, CS23 reduced PA’s mean height (60%) more than UA48 (55%).

CS22 (71%), CS49 (63%), CS50 (80%), TM-1 (68%), and CS34 (70%) had a better PA mean height reduction at 14 DAE than Enlist (59%). Hence, the best- and the worst-performing CS lines/variety in reducing the PA’s mean height at 14 DAE were CS50 and Enlist, respectively. At 21 DAE, UA48 reduced the PA’s mean height by 55%, which was better than CS34 (51%). At 21 DAE, TM1 exhibited the best PA mean height reduction (79%), followed by CS22 (66%), CS50 (66%), and CS34 (63%). All the CS lines outperformed the commercial cultivar, UA48 (55%), in reducing PA’s mean height. The best- and worst-performing cotton lines (PA mean height reduction) at 21 DAE were TM-1 and Enlist, respectively.

In terms of the Palmer amaranth (PA) mean chlorophyll reduction (%) observed over the 21-day period, the CS line CS34 exhibited a remarkable performance, achieving a reduction percentage of 60%, surpassing the performance of conventional cultivars, i.e., TM1 (44%), UA48 (34%), and Enlist (32%). Notably, CS34 outperformed CS50 (52%), CS23 (48%), CS49 (46%), TM1 (44%), and CS22 (40%) in terms of chlorophyll reduction in the PA. On the other hand, Enlist demonstrated the lowest PA mean chlorophyll reduction, at 32%. At the end of the 21 days, considering the PA mean height reduction at 14 DAE (%) and 21 DAE (%), alongside the PA mean chlorophyll reduction (%), the best- and the worst-performing cotton lines were CS 34, CS22, and CS50.

**Figure 3:**
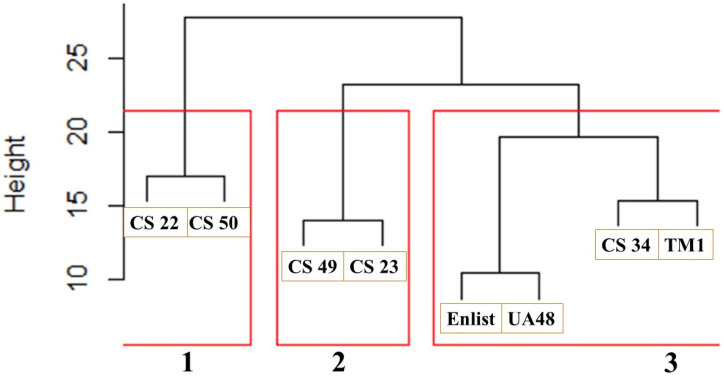
Hierarchical dendrogram illustrating the interrelationships among cotton lines and their respective cluster numbers (red boxes). The dendrogram reveals pairings of the cotton lines determined by their similarities with respect to the measured parameters.

At the end of the 21 days, in relation to the PA mean height reduction (%) at 14 and 21 DAE and the PA mean chlorophyll reduction (%), the best- and worst-performing cotton lines were CS34 and Enlist, respectively. A dendrogram was employed to quantify their degrees of similarity to further elucidate the distinct relationships among the cotton lines under study (Figure 3). The dendrogram was made based on parameters encompassing PA height reduction (%) at 21 DAE and the PA chlorophyll reduction (%). As the dendrogram commenced, each cotton line represented an isolated cluster, forming a comprehensive visual representation of their hierarchical relationships.

The initial pairing of the cotton lines brought CS50 and CS22 together, revealing the highest similarity in their effects on Palmer amaranth (PA) height reduction and chlorophyll content 21 days after establishment (DAE). Following this, CS34 and TM-1 exhibited a similar trend, as did CS23 and CS49. Meanwhile, UA48 and Enlist demonstrated the closest resemblance among the pairs, displaying the most significant similarity. Above a height threshold of 20, the clustering revealed three distinct groups: Cluster 1 (comprising CS50 and CS22), Cluster 2 (including CS49 and CS23), and Cluster 3 (consisting of CS34, TM1, Enlist, and UA48). Analyzing the dendrogram, it becomes evident that Cluster 3 performed well, Cluster 2 performed even better, and Cluster 1 achieved the highest level of performance in reducing the PA’s mean height and chlorophyll content. This hierarchical clustering approach provides valuable insights into the relationships and effectiveness of the cotton lines in terms of the PA height and chlorophyll reduction (%) at 21 DAE.

**Figure 4:**
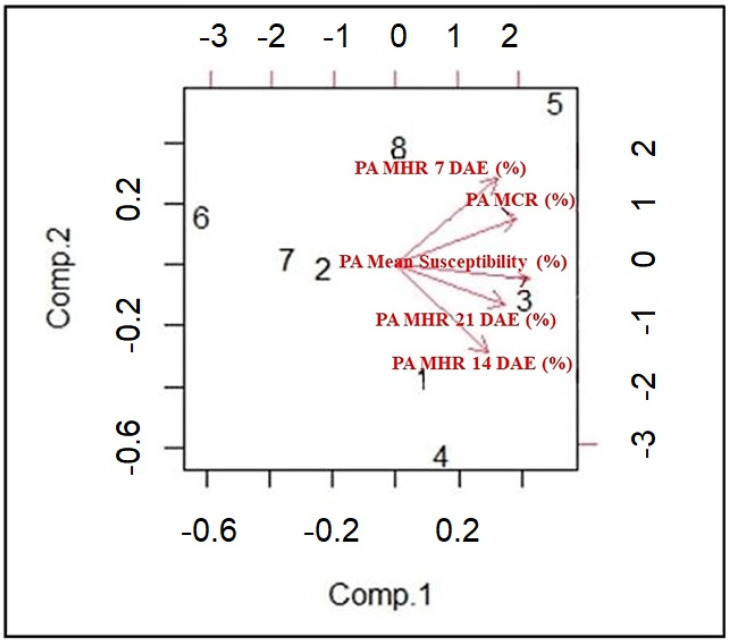
Biplot depicting the interrelationships among CS cotton lines utilizing two components (Components 1 and 2), derived from analyzing five parameters. The five parameters were Palmer amaranth’s mean height reduction (MHR) at 7 DAE (%), mean height reduction (MHR) at 14 DAE (%), mean height reduction (MHR) at 21 DAE (%), mean chlorophyll reduction (MCR) at 21 DAE (%), and mean susceptibility (%). The numbers 1–8 correspond to CS cotton lines, namely: CS22, CS49, CS50, TM1, CS34, Enlist, UA48, and CS23.

To visualize the results of the Principal Component Analysis (PCA), a biplot was created using R software (Figure 4). On the biplot, the axes are ranked in order of their importance. Differences among the cotton lines along the Component 1 axis are much more important than those along that of Component 2. Components 1 and 2 explained 66% and 18% of variation, respectively. From the biplot, while CS22 and TM1 maintained the PA mean height reduction, Enlist, UA48, CS49, and CS23 maintained the PA chlorophyll reduction. CS34 and CS50 had a high PA mean height reduction.

**Figure 5:**
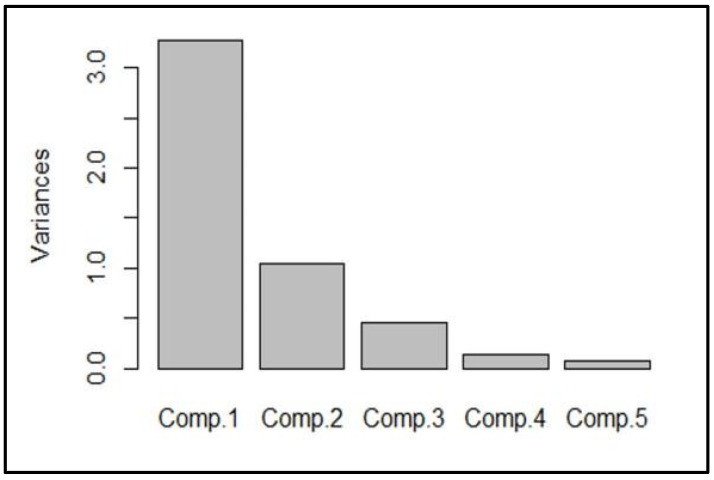
Variability due to five components: cotton height reduction at 7 DAE (%), cotton height reduction at 14 DAE (%), cotton height reduction at 21 DAE (%), cotton chlorophyll reduction (%), and mean susceptibility (%). These components correspond to Components 1, 2, 3, 4, and 5.

Based on the observed variation among the recorded traits, Principal Component Analysis (PCA) was performed (Figure 5). The five components explained the total variance in the weed-reducing traits. Component 1 explained the maximum (above 3.0) of the total variation, while components 2, 3, 4, and 5 explained, at most, 1.0 of the total variation.

**Figure 6:**
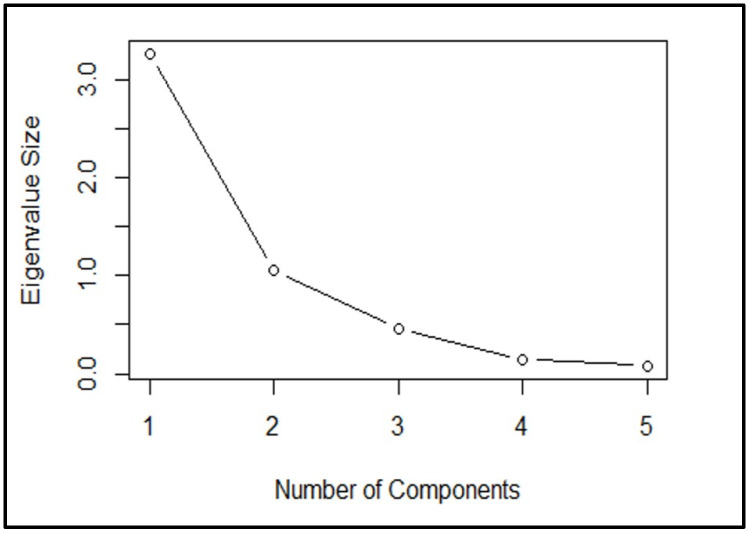
Schematic illustration of a scree plot depicting potential numbers of components determined by the magnitudes of eigenvalues.

The scree plot visually represents the cumulative variance accounted for and elucidated by each principal component, with the components listed on the *x*-axis and the cumulative variance explained on the *y*-axis. According to the scree plot, we interpret the number of components above, where they tend not to change much anymore (Figure 6). The point on the plot where the curve starts to level off or does not change significantly indicates that adding more components does not explain much additional variance in the data. Its purpose is to decide on the number of components or factors to retain. At an eigenvalue (a measure of variance) size greater than 1, where the line starts to curve, there is only one component. However, three components are formed at an eigenvalue size of less than 0.5. This suggests that only one component can be retained if a significant change in the eigenvalues at a certain point, such as when it is greater than 1. On the other hand, if the eigenvalue size is less than 0.5, we need to keep three components.

## 4. Discussion

Weed scientists across the globe have become interested in mitigating the effects of weeds because of their effects on crop production. Eliminating the impacts of weed infestation requires both physical labor and financial resources, which usually causes stress for farmers and economies. The call for pragmatic weed control, stemming from the over-reliance on a particular herbicide leading to the herbicide resistance of weeds, has been echoed during the past decade. Allelopathy is a practical weed control mechanism that can be used to complement herbicide use and ultimately as a stand-alone weed control for integrated weed management [20,21,22]. According to [23], allelopathic weed management can be carried out by either growing allelopathic plants next to weeds that produce these compounds or by planting materials made from allelopathic dead plants in close proximity to weeds. Another way of implementing allelopathic weed control is by growing allelopathic plants in a field for a set amount of time to suppress weeds by releasing allelochemicals from their roots [24].

The plant height and chlorophyll content are important growth aspects of plants. Hence, measuring their reductions was a reliable way of measuring and quantifying the extent to which the weed seedlings were outgrowing the cotton lines. In 2005, Stelly and colleagues achieved success in creating chromosome substitution (CS) cotton lines through the hybridization of *Gossypium hirsutum* (TM-1) with *G. barbadense* and *G. tomentosum* [4]. Using the stairstep structure created an avenue for determining the possible allelopathic effect of the donor plant (cotton lines) on the recipient plant (PA seedlings). In previous studies, the stairstep structure has been used to assess the allelopathic potential of *Oryza sativa* in relation to *Echinochloa crus-galli* [19] and *Gossypium hirsutum* against *A. palmeri* [2] and *Ipomoea batatas* against *A. palmeri* [25].

In research conducted by Wu and colleagues (2009) [26], the CS line B16 displayed a shorter stature than TM-1 plants throughout all stages of growth. This finding suggests that chromosome 16 in *G. barbadense* contributes to reduced plant height. In another study by Awasthi and coworkers (2018) [27], CS-Bo2 stood out with a notable 155% increase in height, indicating the potential influence of genes associated with the substituted chromosome 2 from *G. barbadense*.

When faced with stress, plants direct their energy to defense or growth [28], and allelopathy is known to be affected by environmental stressors [29]. In this study, CS 34, CS50, and CS22 performed better than the other CS lines and conventional varieties at the end of the 21 days. These heat-tolerant lines from *G. tomentosum* could suppress the height and chlorophyll concentration of Palmer amaranth. *G. tomentosum* (Hawaiian cotton) is regarded as one of the most heat-tolerant species within the genus, which could be because of the dry and rocky nature of its coastal habitats (Percival et al., 1999; Brubaker et al., 1994) [30,31]. This could be the reason for the better performance of CS lines derived from *G. tomentosum* (CS50 and CS22) compared to the conventional varieties and the other CS lines derived from *G. barbadense*. In previous research, Fuller and coworkers (2021) [2] reported that CS50 (T26lo) reduced the height and chlorophyll concentrations of PA. A reduction in chlorophyll affects photosynthesis, ultimately impacting a plant’s overall growth (Singh et al., 2004) [32]. A reduction in chlorophyll concentrations has been reported in several weed suppression studies. In a study on the use of monoterpenes to suppress weeds, researchers reported a reduction in the chlorophyll concentration of weeds (*E. crus-galli* and *Bidens pilosa*) by monoterpenes (Singh et al., 2004) [32]. Phenolic allelochemicals modify enzyme activity and function by penetrating the plant cells, which, in turn, reduces the oxygen absorption capacity, impacts respiration rates, lowers the chlorophyll content, and ultimately leads to decreased photosynthesis rates (Gurmani et al., 2021) [33].

PCA and clustering have been used to group allelopathic weed-suppressive cotton lines based on their weed-suppressive potential, which can aid in gaining a deeper insight into breeding material for crop enhancement (Fuller et al., 2021) [2]. Similarly, the grouping of rice cultivars by Schumaker et al. (2020) [19] and sweet potato varieties by Singh et al. (2022) [25] has been carried out using similar strategies. Allelopathy often occurs as a response to challenging conditions, as noted by Kruse and a group of researchers in their study published in 2000. The results of our research demonstrated that CS22, CS34, and CS50 exhibit characteristics ranking among the top-performing cotton lines in terms of reducing the height and chlorophyll concentration of Palmer amaranth. These specific CS lines carry alleles from *G. barbadense* and *G. tomentosum*, both species known for being more susceptible to stress compared to *G. hirsutum* on its own (Shavkiev et al., 2022) [34].

The production of allelochemicals Is influenced by environmental factors and genetic traits (Xiong et al., 2007) [35]. Therefore, the activation of genes responsible for allelopathic effects is significantly influenced by specific environmental conditions employed in experiments (Kruse et al., 2000) [29]. In our study, using a stairstep structure and a controlled greenhouse environment has created optimal conditions for assessing the weed-suppressing capabilities of various cotton lines on Palmer amaranth. However, testing these lines in an agronomic setting under natural environmental conditions would provide valuable insights into the real-world performance and suitability of these cotton lines.

## 5. Conclusions

The findings from this study illuminate the physiological and genetic mechanisms underlying weed suppression in cotton genotypes, particularly in the presence of Palmer amaranth. CS lines CS22, CS23, CS34, and CS50 exhibited superior growth and chlorophyll formation traits, suggesting their competitive advantage against Palmer amaranth. These results imply a genetic basis for weed suppression, potentially associated with specific chromosomes or genetic segments inherited from *G. barbadense* and *G. tomentosum*, such as chromosome 16 and chromosome 2. Further exploration of the allelopathic compounds and metabolic pathways linked to these strains is warranted. Understanding the genetic determinants of weed suppression in cotton will inform targeted breeding efforts and facilitate sustainable weed management strategies in agriculture.

## Figures and Tables

**Table 1:** Palmer amaranth height and chlorophyll concentration reduction values.

Accession	PA Mean Height Reduction 14 DAE (%)	PA Mean Height Reduction 21 DAE (%)	PA Mean Chlorophyll Reduction (%)	Mean Susceptibility (%)
CS 22 (T11sh)	70.6 ^ab^	66.4 ^ab^	39.67 ^bc^	58.89 ^ab^
CS 49 (B26lo)	63 ^ab^	59.38 ^bc^	46.22 ^bc^	48.73 ^bc^
CS 50 (T26lo)	79.19 ^a^	65.61 ^ab^	51.49 ^ab^	58.61 ^ab^
TM1	68.44 ^ab^	78.81 ^a^	44.37 ^bc^	55.16 ^ab^
CS 34 (BNTN)	70.32 ^ab^	63.09 ^ab^	60.07 ^a^	60.72 ^a^
Enlist	59.16 ^b^	50.79 ^c^	32.24 ^d^	42.25 ^c^
UA48	66.6 ^ab^	54.97 ^bc^	33.61 ^cd^	46.49 ^bc^
CS 23 (B10)	62.21 ^ab^	60.4 ^b^	48.02 ^b^	52.89 ^b^

Mean height reduction of PA at 14 and 21 DAE and chlorophyll reduction at 21 DAE. The mean susceptibility was derived from Palmer amaranth height and chlorophyll reduction by each CS line. The means were separated using Student’s *t*-test at α = 0.05. Based on their significance level, the linked groups are marked with corresponding letters.

## Data Availability

Dataset available on request from the authors.
